# Transcription factors regulating vasculogenesis and angiogenesis

**DOI:** 10.1002/dvdy.575

**Published:** 2023-03-06

**Authors:** Sophie Payne, Alice Neal, Sarah De Val

**Affiliations:** ^1^ Department of Physiology, Anatomy and Genetics Institute of Developmental and Regenerative Medicine, University of Oxford Oxford UK

**Keywords:** endothelial cell, angioblast, transcriptional regulation, vasculature, endothelium, ETS

## Abstract

Transcription factors (TFs) play a crucial role in regulating the dynamic and precise patterns of gene expression required for the initial specification of endothelial cells (ECs), and during endothelial growth and differentiation. While sharing many core features, ECs can be highly heterogeneous. Differential gene expression between ECs is essential to pattern the hierarchical vascular network into arteries, veins and capillaries, to drive angiogenic growth of new vessels, and to direct specialization in response to local signals. Unlike many other cell types, ECs have no single master regulator, instead relying on differing combinations of a necessarily limited repertoire of TFs to achieve tight spatial and temporal activation and repression of gene expression. Here, we will discuss the cohort of TFs known to be involved in directing gene expression during different stages of mammalian vasculogenesis and angiogenesis, with a primary focus on development.

## Key Findings

- Up‐to‐date summary of all transcription factors involved in angiogenesis and vasculogenesis Discusses the roles for each factor in the vasculature, including the consequences of transcription factor ablation, and known downstream targets of each factor Includes comprehensive tables listing consensus binding motifs, expression profiles and available ChIP‐seq datasets for all endothelial transcription factors alongside known enhancers with bound transcription factors. This provides an essential and much needed resource for all scientists researching vascular biology, particularly in the era of single cell ATAC and RNA‐seq datasets

## 1 INTRODUCTION

In the early mammalian embryo, endothelial cells (ECs) are formed de novo through a process known as vasculogenesis, canonically defined as the differentiation of specific mesodermal precursors into endothelial progenitor cells known as angioblasts. This may occur via multipotential blood islands, although the existence of a shared progenitor population for hematopoietic and endothelial lineages remains subject to debate. Hemato‐endothelial progenitor cells can be detected in the mouse from embryonic days (E) 6.75–7.0, with angioblasts identified within the lateral plate mesoderm by E7.5.^1^, ^2^, ^3^, ^4^, ^5^, ^6^ By E8.0 angioblasts have begun to form the first vascular structures, organizing into two parallel tracts to form the primitive paired dorsal aorta, and along the cardiac crescent as a precursor to the endocardium.^5^ Vein primordia form soon after at the yolk sac/embryo interface, presaging the future sinus venosus, and by E8.5 the first intra‐embryonic veins coalesce and a primitive vascular plexus can be found throughout the embryo.^5^

Once the vascular plexus has been established, subsequent growth of blood vessels in the embryo and in the adult primarily occurs via angiogenesis. While angiogenesis by definition refers to the formation of blood vessels from existing ones via any mechanism, the best described and apparently most abundant form of angiogeniesis is sprouting angiogenesis. In this dynamic process, a subset of ECs within the pre‐existing vasculature responds to growth factors such as VEGF‐A (via VEGFR2) and CXCL12 (via CXCR4) to become filopodia‐rich migratory tip cells and invade the interstitial space.^7^, ^8^, ^9^ In turn, tip cells use DLL4/JAG‐Notch signaling to suppress migratory behavior in neighboring ECs, which instead become highly proliferative stalk cells, enabling vessel elongation and lumen formation.^10^ Further, tip cells with high Notch activity can also migrate toward, and differentiate into, arterial ECs.^9^ In addition to VEGF‐VEGFR2, CXCL12‐CXCR4, and NOTCH signaling, multiple additional growth factors and signaling pathways provide both positive and negative regulatory inputs to control angiogenesis, as do cell adhesion interactions with the extracellular matrix. Thus sprouting angiogenesis allows a vascular bed to expand in response to hypoxia and insufficient nutrients, both during development and during physiological processes in the adult such as wound healing and changes in the lining of the endometrium. Pathological angiogenesis can also contribute to and aid the development of conditions such as cancer, atherosclerosis, and age‐related macular degeneration.

The transition from mesodermal progenitors into a differentiated, functional endothelium depends on the activity of lineage‐defining genes. Similarly, the coordinated response of ECs to the complex signals directing sprouting angiogenesis is dependent on the tight spatio‐temporal expression and repression of specific cohorts of genes. Transcription factors (TFs) play a key role in this dynamic gene regulation. These DNA‐binding proteins influence transcription by binding to specific motifs within cis‐regulatory elements (enhancers and promoters), in combinations that often include both lineage‐specifying factors (e.g. ETS factors for ECs) and transcriptional effectors of different signaling pathways (e.g., RBPJ for Notch, SMADs for TGFβ/BMP). In this way, multiple different environmental cues can collectively influence enhancer/promoter activity and subsequent gene expression.^11^, ^12^ While most TFs have a defined “consensus” DNA binding motif, usually established using either ChIP‐seq (chromatin immunoprecipitation combined with sequencing) or HT‐SELEX (high‐throughput systematic evolution of ligands by exponential enrichment), TFs are also often able to bind slightly alternative (non‐consensus) sequences, a process that can be influenced by other bound or accessory proteins and that likely also contributes to the differing patterns of gene activation downstream of different enhancers. In this review, we aim to summarize what is currently understood about the complex network of TFs that orchestrate the formation of a functional circulatory network in the mammalian embryo through the processes of vasculogenesis and sprouting angiogenesis. In addition, we provide three tables covering specific aspects of this regulation, including the known binding motifs for each endothelial TF (Table 1), the expression patterns of each TF during mammalian embryonic development and available ChIP‐seq data sets (Table 2), and a detailed assessment of all in vivo‐characterized mammalian endothelial enhancers and their cognate binding TFs (Table 3).

**TABLE 1:** Consensus binding motifs for common EC transcription factors (TFs)

TF	Sequence logo	Type	Species/cell type	Source	TF	Sequence logo	Type	Species/cell type	Source
ETV2	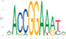	HT‐SELEX	Human Transfected LoVo/Hek293	JASPAR^13^, ^14^	GATA/TAL1	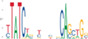	ChIP‐seq	Mouse Erythroid progenitors	Primary publication^15^
ETV2	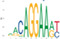	ChIP‐seq	Mouse Differentiated ES cell	Primary publication^1^	TAL1	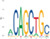	ChIP‐seq	Mouse Erythroid progenitors	Primary publication^15^
FLI1	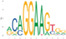	ChIP‐seq	Human Not specified	JASPAR/PAZAR^14^, ^16^	SMAD4	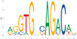	ChIP‐seq	Human Unspecified	JASPAR2016/PAZAR^16^, ^17^
FLI1	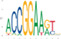	HT‐SELEX	Human Transfected LoVo/Hek293	JASPAR^13^, ^14^	SMAD4	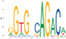	ChIP‐seq	Human Embryonic stem cells	Primary publication^18^
FLI1	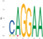	ChIP‐seq	**Human** **HUVEC**	Primary publication^19^	SMAD1 SMAD5	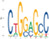	ChIP‐seq	**Human** **HUVEC**	Primary publication^20^
ERG	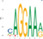	ChIP‐seq	**Human** **HUVEC**	Primary publication^19^	HIF1A	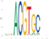	ChIP‐seq	Human Not specified	JASPAR/ReMap2018^14^, ^21^
ERG	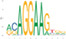	ChIP‐seq	Mouse Not specified	JASPAR/PAZAR^14^, ^16^	HIF1A	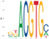	ChIP‐seq	**Human** **HUVEC**	Primary publication^22^
ETS1	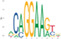	ChIP‐seq	**Human** **HUVEC**	Primary publication^23^	RBPJ	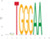	ChIP‐seq	Human Not specified	JASPAR/ReMap2018^14^, ^21^
ETS1	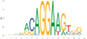	ChIP‐seq	Mouse Not specified	JASPAR/PAZAR^14^, ^16^	HEY1	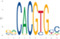	ChIP‐seq	Human Hek293, Mouse hearts	JASPAR^13^, ^14^
ELK3	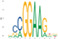	ChIP‐seq	**Human** **HUVEC**	Primary publication^24^	MEF2A	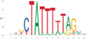	ChIP‐seq	Human Not specified	JASPAR/ReMap2018^14^, ^21^
ELK3	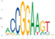	HT‐SELEX	Human Transfected LoVo/Hek293	JASPAR^13^, ^14^	MEF2C	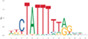	ChIP‐seq	Human Not specified	JASPAR/ENCODE^14^
FOXC1	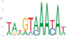	HT‐SELEX	Human Transfected LoVo/Hek293	JASPAR^13^, ^14^	JUNB	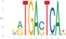	ChIP‐seq	Human Not specified	JASPAR/ENCODE^14^
FOXC2	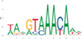	HT‐SELEX	Human Transfected LoVo/Hek293	JASPAR^13^, ^14^	MAFB	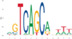	ChIP‐seq	Mouse Not specified	JASPAR^14^
FOXC2	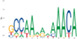	ChIP‐chip	**Human** **Lymphatic ECs**	Primary publication^25^	MAFF	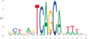	ChIP‐seq	Human Not specified	JASPAR/ENCODE^14^
FOXO1	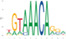	ChIP‐seq	Mouse Not specified	JASPAR/PAZAR^14^, ^16^	TEAD1	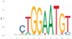	ChIP‐seq	Human Not specified	JASPAR/ReMap2022^14^, ^26^
FOXP1	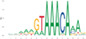	ChIP‐seq	Human Not specified	JASPAR/ PAZAR 228,230	SOX7	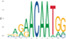	ChIP‐seq	Mouse ESC‐embryoid body	Primary publication^27^
GATA2	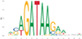	ChIP‐seq	Human Not specified	JASPAR/ENCODE^14^	SOX17	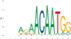	ChIP‐seq	Mouse Not specified	JASPAR/ReMap2022^14^, ^26^
GATA2	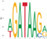	ChIP‐seq	**Human** **HUVEC**	Primary publication^28^	SOX18	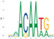	ChIP‐seq	**Human** **HUVEC**	Primary publication^29^

*Note*: JASPAR refers to JASPAR 2022 unless specified, logos from primary publications are adapted by authors from the original paper. ChIP‐seq refers to chromatin immunoprecipitation combined with sequencing; HT‐SELEX refers to high‐throughput systematic evolution of ligands by exponential enrichment, bold denotes that ECs were experimental model used in motif designation. Note that consensus motifs should be viewed as only a guide to potential TF binding locations, as TFs can also bind non‐consensus motifs (often similar to consensus) and conversely do not always bind sequences containing the exact consensus motif.

**TABLE 2:** Expression dynamics of common endothelial TFs and information on publicly deposited ChIP‐seq data sets

Transcription factor gene	**Earliest time‐point expressed in hemato‐endothelial progenitors (from** ^**2**^ **)** *= expression in most cells	Cell type with highest expression of TF in E9–E13 embryos (from ^30^)	**Other tissues expressing TF** List of next five highest cell types expressing as indicated and defined by Mouse Atlas data set,^30^ italics indicate expression is <50% of EC level, cut off is 10% of EC expression	**Chip‐seq data from ECs, progenitor cells or whole tissues** Cell type, GEO accession number, reference
*Etv2*	E6.75	Not expressed	Not expressed at detectable levels	In‐vitro differentiated ES cells expressing inducible ETV2‐V5 GSE5940^1^
*Fli1*	E6.75–E7.0	**Endothelial cells**	Megakaryocytes, white blood cells, jaw/tooth progenitors*, early mesenchyme, limb mesenchyme*	HUVECs with and without VEGFA stimulation, GSE109625^31^; HUVECs with and without ERG and FLI siRNA, GSE109695^19^
*Erg*	E7.75	**Endothelial cells**	*Jaw/tooth progenitors, chondrocytes/osteoblasts, limb mesenchyme, osteoblasts, neutrophils*	HUVECs with and without VEGFA stimulation, GSE109625^31^; HUVECs and HUAEC, GSE128382^32^; HUVECs GSE124893^33^; HAECs under control or inflammatory stimuli, GSE89970^34^
*Ets1*	E7.25	**Endothelial cells**	*Melanocytes, chondrocyte progenitors, Schwann cell precursors, intermediate mesoderm, connective tissue progenitors*	HUVECs with and without VEGFA stimulation, GSE109625^31^; HUVECs with and without VEGFA stimulation GSE93030^23^
*Ets2*	E7.25*	**Endothelial cells**	Limb mesenchyme, *stromal cells, early mesenchyme, osteoblast, cardiac muscle lineages*	
*Elk3*	E7.0	**Endothelial cells**	*Limb mesenchyme, white blood cells, jaw/tooth progenitors, early mesenchyme, chondrocytes/osteoblasts*	HUVECs in normoxia, GSE60156^24^
*Foxc1*	E8.25	Chondrocyte progenitors	Jaw/tooth progenitors, stromal cells, osteoblasts, early mesenchyme, **endothelial cells**	
*Foxc2*	E7.75	Chondrocyte progenitors	Jaw/tooth progenitors, stromal cells, osteoblasts, early mesenchyme, **endothelial cells**	
*Foxo1*	E7.75	**Endothelial cells**	Myocytes, *Schwann cell precursor*, *hepatocytes*, *chondrocyte progenitors*, *epithelial cells*	HUVECs with or without constitutively active FOXO1, GSE128635^35^; mouse heart after isoproterenol, transverse aortic constriction or vehicle/sham, GSE144011^36^
*Foxo3*	N/A	Primary erythroid lineage	Definitive erythroid lineage, white blood cells, megakaryocytes, hepatocytes, intermediate mesoderm	
*Foxo4*	N/A	Primary erythroid lineage	Myocytes, cardiac muscle lineages, definitive erythroid lineage, lens, osteoblasts	
*Foxm1*	E7.0*	Premature oligodendrocyte,	Oligodendrocyte progenitors, neural tube, radial glia, isthmic organizer cells, primitive erythroid lineage.	
*Gata2*	E7.0	Megakaryocytes	White blood cells, inhibitory neuron progenitors, **endothelial cells**, neural progenitor cells, intermediate mesoderm	HUVECs, GSE29531^28^; HUVECs GSM935347, as part of 37
*Gata3*	E7.0	Inhibitory neuron progenitors,	Inhibitory neurons, epithelial cells, lens, neutrophils, excitatory neurons	
*Gata6*	E7.0	Intermediate mesoderm	Cardiac muscle lineages, hepatocytes**, endothelial cells,** stromal cells, connective tissue progenitors	
*Tal1*	E7.0	Primitive erythroid lineage	Definitive erythroid lineage, megakaryocytes, **endothelial cells,** inhibitory neuron progenitors, white blood cells	
*Smad1*	E7.0*	Sensory neurons	**Endothelial cells,** neural progenitor cells, stromal cells, postmitotic premature neurons	HUVECs with BMP6/9 stimulation, GSE27661^20^
*Smad2*	E7.0*	Stromal cells	*Limb mesenchyme, early mesenchyme, Schwann cell precursor, hepatocytes, neural tube*	
*Smad3*	E8.25*	Epithelial cells	Isthmic organizer cells, chondrocyte progenitors, stromal cells, limb mesenchyme	
*Smad4*	E6.75*	Stromal cells	Early mesenchyme, limb mesenchyme, neural tube, neural progenitor cells, cardiac muscle lineages	
*Smad5*	E7.0*	Limb mesenchyme	Stromal cells, **endothelial cells,** melanocytes, chondrocyte/osteoblasts, osteoblasts	HUVECs with BMP6/9 stimulation, GSE27661^20^
*Smad6*	E7.0	**Endothelial cells**	Intermediate mesoderm, cardiac muscle lineages, stromal cells, epithelial cells, limb mesenchyme	
*Smad7*	E6.75*	Stromal cells	Limb mesenchyme, **endothelial cells**, intermediate mesoderm, chondrocytes/osteoblast, cardiac muscle lineage	
*Smad9*	N/A	Intermediate mesoderm	Jaw and tooth progenitors, neutrophils, cardia muscle linages, ependymal cell, osteoblasts	
*Hif1a*	E7.0*	Cardiac muscle lineages	Stromal cells, melanocytes, early mesenchyme, chondrocytes/osteoblasts, jaw and tooth progenitors	HUVEC under normoxia and hypoxia, GSE39089,^22^ HUVECs grown in hypoxia, GSE89836^38^
*Hif1b*	E7.5*	Connective tissue progenitors	Chondrocyte progenitors, primitive erythroid lineage, stromal cells, definitive erythroid lineage, hepatocytes	HUVECs grown in hypoxia, GSE89836^38^
*Hif2a*	E8.5	**Endothelial cells**	Hepatocytes, chondrocyte progenitors, intermediate mesoderm, osteoblasts, megakaryocytes	HUVECs grown in hypoxia, GSE89836^38^
*Rbpj*	E7.0*	White blood cells	Neural progenitor cells, myocytes, stromal cells, granule neurons, neural tube	HUVECs with and without VEGFA stimulation, GSE109625^31^; quiescent and confluent HUVECs, GSE85987^39^
*Notch1*	E7.0	Radial glia	Neural tube, oligodendrocyte progenitors, **endothelial cells**, neural progenitor cells, Schwann cell precursor	Quiescent and confluent HUVECs, GSE85987^39^
*Notch4*	E8.25	**Endothelial cells**	Chondrocyte progenitors, osteoblasts, lens, cardiac muscle lineages, stromal cells	
*Hey1*	E7.75	Notocord cells	**Endothelial cells**, *premature oligodendrocyte, myocytes, Schwann cell precursor, limb mesenchyme*	
*Hey2*	E8.0	Schwann cell precursor	Cardiac muscle lineages, chondrocyte progenitors, chondrocytes/osteoblasts, **endothelial cells**, premature oligodendrocyte	
*Hlx*	E7.75	**Endothelial cells**	Intermediate mesoderm, neutrophils, white blood cells, *lens, connective tissue progenitors*	
*Mef2a*	E7.5*	**Endothelial cells**	Myocytes, white blood cells, cardiac muscle lineages, chondrocyte progenitors, connective tissue progenitors	Mouse hearts, GSE124008^40^
*Mef2c*	E7.75	White blood cells	**Endothelial cells,** myocytes, Schwann cell precursor, megakaryocytes, cardiac muscle lineages	HUVECs with and without statins, GSE32644^41^; Mouse hearts, GSE124008^40^
*Mef2d*	E7.75	**Endothelial cells**	Myocytes, hepatocytes, *white blood cells, primitive erythroid lineage, definitive erythroid lineage*	
*Junb*	E7.0	Primitive erythroid lineage	**Endothelial cells,** definitive erythroid lineage, white blood cells, hepatocytes, megakaryocytes	HAECs under control or inflammatory stimuli, GSE89970^34^
*Jun*	E6.75*	Osteoblasts	Cholinergic neurons, cardiac muscle lineages, **endothelial cells**, neutrophils, stromal cells	HAECs under control or inflammatory stimuli, GSE89970^34^
*Mafb*	E8.5	White blood cells	Cholinergic neurons, ependymal cells, osteoblasts, neutrophils, inhibitory neurons	
*Maff*	E7.75	Hepatocytes	**Endothelial cells,** lens, *cardiac muscle lineages, stromal cells, epithelial cells*	
*Mafg*	E7.0*	Primitive erythroid lineage	White blood cells, definitive erythroid lineage, megakaryocytes, hepatocytes, lens	
*Mafk*	E8.5	Primitive erythroid lineage	Definitive erythroid lineage, megakaryocytes, white blood cells, **endothelial cells**, cardiac muscle lineages	
*Tead1*	E7.5*	Ependymal cells	Epithelial cells, notochord cells, neural tube, radial glia, melanocytes	Mouse hearts, GSE124008^40^
*Tead2*	E6.75*	Stromal cells	Limb mesenchyme, early mesenchyme, premature oligodendrocyte, chondrocyte/osteoblasts, oligodendrocyte progenitors	
*Tead4*	E7.75	Myocytes	**Endothelial cells,** epithelial cells, intermediate mesoderm, melanocytes, chondrocyte progenitors	
*Sox7*	E7.0	**Endothelial cells**		HUVECs overexpressing SOX7‐mCherry, ChIP of mCherry E‐MTAB‐4480 (ArrayExpress database)^29^
*Sox17*	E7.75	**Endothelial cells**		
*Sox18*	E7.75	**Endothelial cells**	*Osteoblasts, cardiac muscle lineages*	HUVECs overexpressing SOX18‐mCherry, ChIP of mCherry E‐MTAB‐4481 (ArrayExpress database)^29^

**TABLE 3:** A list of in vivo‐validated EC enhancers alongside approximate location and associated regulatory TFs

Gene (species)	Enhancer name in original paper	Enhancer location	Validation method	EC specificity (age investigated)	Transcription factors with experimental validation method	References
***Acvrl1/Alk1* (*ms*)**	Alk1 pXh4.5‐in2‐SIB	Promoter/intronic −3 to +6 kb (9 kb piece)	Tg mouse	Arterial EC (E11.5‐adult)	**SP1**: mo **AP2:** mo **NFKB:** mo **GATA:** mo **CREB:** mo **ETS:** mo	42, 43
***Apln* (*ms*)**	Apln	Downstream +28 kb	Tg mouse	Pan‐EC (E11.5)		44
***CDH5/VE‐cadherin* (*hm/ms*)**	hVEcad promoter	Upstream to promoter −1 kb to TSS	Tg mouse	Pan‐EC (E7.5–E12.5/adult)	**FOXC/O**: mo, em, ch, oe/OE, MU **ETS**: mo, em (ETS1), oe/OE (ETV2), MU **GATA**: mo, oe, mu **TAL1:** mo, oe, mu	45, 46, 47
***Dab2* (*ms*)**	Dab2	Upstream −240 kb	Tg mouse	Pan‐EC (E11.5)		44
***Dll4* (*ms*)**	Dll4in3/Dll4‐F2	Intronic +2 kb	Tg mouse Tg zebrafish	Arterial EC Angiogenic EC (E8–P8, 24–72 hpf)	**ETS**: mo, em (ETS1/ERG), ch (ERG), mu/MU, KD (ERG) **SOXF**: mo, ch (SOX7/18), em (SOX7/18), MU, KD (*sox7/sox18*) **RBPJ**: mo, ch, em, MU, KD NOTCH: ch **MEF2**: mo, chs (MEF2C), em (MEF2A/C/D), MU, KD/KO **KLF4:** mo, ch, oe **B‐catenin:** ch, oe	48, 49, 50, 51, 52, 53, 54, 55
***Dll4* (*ms*)**	Dll4‐12	Upstream −12 kb	Tg mouse	Arterial EC (E8–P8)	**SOXF**: mo, ch (SOX7/18), em (SOX7/18), MU, KD (*sox7/sox18*) **RBPJ**: mo, ch, em, MU, KD **ETS** (all): mo	50, 54
***ECE1* (*hm*)**	ECE1 enhancer	Promoter or intronic +10 kb^a^	Tg mouse	Arterial EC (E7.75–E12.5)	**FOXC/O**: mo, em (FOXC2), oe, MU **ETS**: mo, em (ETV2), oe, MU **SOXF**: mo, em (SOX17), oe, mu, MU	45, 56
***Egfl7* (*ms*)**	Egfl7_E1	Upstream −9 kb	Tg mouse	Pan‐EC (E11.5)		44
***Egfl7* (*ms*)**	Egfl7_E3	Upstream ‐2 kb	Tg mouse	Pan‐EC (E11.5)		44
***EMCN* (*hm*)**	EMCN‐22	Upstream −22 kb	Tg mouse	Venous EC (E11.5)	**ETS**: mo **SMAD**: mo, chs (SMAD1/5)	57, 58
***Eng* (*ms*)**	Eng‐8	Upstream −8 kb	Tg mouse	Pan‐EC (E11.5)	**ETS**: mo, ch (FLI1/ERG/ELF1), mu/MU	59
***Eng* (*ms*)**	End+9	Intronic +9 kb	Tg mouse	EC^a^ (E11.5)	**ETS:** mo, ch (FLI1) MU	60
***Ephb4* (*ms*)**	Ephb4‐2	Upstream −2 kb	Tg mouse Tg zebrafish	Venous EC (E9–E15.5, 24–72 hpf)	**ETS**: mo, em (ETS1/ERG), ch (ERG), chs (ETS1/ERG), MU, KD **SMAD**: mo, ch (SMAD1/5), chs (SMAD1/5), MU, KO	44, 57, 58
***Etv2* (*ms*)**	Etv2‐enhancer	Upstream to promoter −3 kb to TSS	Tg mouse	Early ECs (E7.75–E8.75)	**ETS**: mo, mu, oe **NFAT**: mo, oe **SMAD**: mo **GATA**: mo, oe **MESP/CREB:** mo, mu, oe **NKX2‐5:** mo, em, ch	61, 62, 63, 64, 65
***Fli1* (*ms*)**	Fli1+12	Intronic +12 kb	Tg mouse	Pan‐EC (E9.5–E12.5)	**GATA**: mo, em, ch (GATA2) **ETS**: mo, mu/MU, ch (FLI1/ELF) **TAL1**: mo (Ebox), ch	66, 67
***Flk1* (*ms*)**	Flk1 minimal enhancer/Flk1in1	Intronic +3.5 kb	Tg mouse	Pan‐EC (E11.5)	**ETS**: mo, em (ETS1), oe/OE (ETV2) **GATA**: mo, em (GATA2), MU **TAL1**: mo, em, MU **FOXC/O**: mo, em, ch, oe/OE	45, 68
***Flk1* (*ms*)**	Flk1in10	Intronic +16 kb	Tg mouse Tg zebrafish	Pan‐EC (E9.5), Arterial EC (E10–E16)	**ETS**: mo, em (ETV2), MU **GATA**: mo, em (GATA2), MU, KD **SOXF**: mo, em (SOX7), MU **RBPJ**: mo, em, MU, KD, MU **FOXC**: mo, em (FOXC2)	69
***FLT4/VEGFR3* (*hm*)**	FLT4 enhancer/In11‐12TBE	Intronic +26 kb	Tg mouse	Pan‐EC (E9.5) Lymphatic EC (E15.5) Arterial EC (E15.5)	**FOXC/O**: mo **ETS**: mo **TBX1:** mo, mu, ch, oe	45, 70
***FOXP1* (*hm*)**	FOXP1 enhancer	Intronic +138 kb^a^	Tg mouse	Pan‐EC tail region (E9.5)	**FOXC/O**: mo **ETS**: mo	45
***Gata2* (*ms*)**	G2‐EHRD G2‐5H G2‐D3.1	Upstream −3 kb	Tg mouse	EC^a^ (E9.5) Hemogenic EC (E10.5–E11.5)	**GATA**: mo, em, ch (GATA2) **ETS**: mo, mu/MU, ch (FLI1) **TAL1**: mo (Ebox), mu, ch	66, 71
***Gata2* (*ms/zf*)**	Gata2intron4 Gata2‐TKVE Gata2+9.5	Intronic +9 kb	Tg mouse Tg zebrafish	Pan‐EC (E10.5, 27 hpf)	**GATA**: mo **ETS**: mo, MU^b^ **TAL1**: mo, em, MU **MEF2**: mo **SMAD**: mo	72, 73, 74
***Hey1* (*ms*)**	Hey1‐18k	Upstream −18 kb	Tg mouse	Arterial EC (E9.5–10.5)	**RBPJ**: mo, em, MU, oe	75
***HLX* (*hm*)**	HLX‐3/HLX‐3b	Upstream −3 kb	Tg mouse Tg zebrafish	Angiogenic ECs (E11.5, 27–42 hpf)	**ETS**: mo, chs (ERG), mu, MU **MEF2**: mo, MU	49, 51
***Mef2* (*ms*)**	Mef2F10	Intronic +79 kb^a^	Tg mouse Tg zebrafish	Pan‐EC (E9.5, 48 hpf); Venous EC (E11.5)	**ETS**: mo, em (ETS1/ETV2) mu/MU, oe/OE (ETV2) **FOXC/O**: mo, em (FOXC1/2, FOXO1) ch (FOXC2), mu/MU, oe/OE (FOXC2)	44, 45
***Mef2* (*ms*)**	Mef2F7	Intronic +68 kb^a^	Tg mouse	Pan‐EC (E7.5‐adult)	**ETS**: mo, em (ETS1), MU	76
***NOS3/eNOS* (*hm/ms*)**	eNOSprom	Upstream to promoter −5 kb to TSS		Arterial EC (E11.5–E15.5)	**KLF2**: mo, oe, em **GATA**: mo **AP2**: mo, em **MZF‐like**: mo, em **ETS1**: mo, em (ERG), mu	77, 78, 79, 80, 81
***NOTCH1* (*hm*)**	NOTCH1+16	Intronic +16 kb	Tg mouse	Arterial EC (E9.5–E12.5)	**ETS**: mo, em (ETV2) **SOXF**: mo, em (SOX7/18), MU	82
***notch1b* (*zf*)**	notch1b‐15	Upstream −15 kb	Tg zebrafish	Arterial EC (28–48 hpf)	**ETS**: mo, em (ETV2) **SOXF**: mo, em (SOX7/18), MU, KD	82
***NOTCH1* (*hm and ms*)**	NOTCH1+33/Notch1_enh1	Intronic +33 kb	Tg mouse	Pan‐EC (E11–E13)		44, 82
***NOTCH4* (*hm*)**	NOTCH4proIN1	Promoter/intronic +0.7 kb^a^	Tg mouse	EC^a^ (E10.5)	**AP1**: em (FRA1), ch (all‐Fos/FRA1), mu/MU **FOXC/O**: mo, em, ch, oe/OE **ETS**: mo, em (ETS1), oe/OE (ETV2)	45, 83
***Nr2f2/Coup‐TFII* (*ms*)**	CoupTFII‐965	Upstream −965 kb	Tg mouse Tg zebrafish	Venous EC Lymphatic EC (E9–E15.5, 24–72 hpf)	**ETS**: mo, em (ETS1/ERG), ch (ERG), chs (ETS1/ERG), MU, KD **SMAD**: mo, ch (SMAD1/5), chs (SMAD1/5), MU, KO	57, 58
***NRP1* (*hm*)**	NRP1 enhancer	Intronic +32 kb	Tg mouse	Pan‐EC (E9.5)	**FOXC/O**: mo **ETS**: mo	45
***Nrp2* (*ms*)**	Nrp2+26	Intronic +26 kb	Tg mouse	ECs around neural tube^a^ (E11.5)	**ETS**: mo **SMAD**: mo, chs (SMAD1/5)	57, 58
***PDGFRB* (*hm*)**	PDGFRB enhancer	Intronic +18 kb	Tg mouse	Pan‐EC (E9.5)	**FOXC/O**: mo **ETS**: mo	45
***Procr/Epcr* (*ms*)**	‐5.5HS	Upstream −5 kb		Pan‐EC (E12.5)	**ETS**: mo, mu, ch (ETS1/ELF1/FLI1/ERG) **GATA**: mo, mu, ch (GATA2) **TAL1**: mo, mu, ch	84
***PROX1* (*hm/ms*)**	PROX1‐11	Upstream −11 kb	Tg mouse	Lymphatic ECs, particularly in valves (E12–P4)	**ETS:** mo **GATA:** mo, ch and chs (GATA2) **FOXC:** mo, ch (FOXC2) **NFAT:** mo, ch (NFATc1) **AP1:** mo	85, 86
***Sema6d* (*ms*)**	Sema6d	Upstream −55 kb	Tg mouse	Arterial ECs (E11.5)		44
***SOX7* (*hm and ms*)**	Sox7/CRE3	Upstream −14 kb (ms) −10 kb (hm)	Tg mouse Tg zebrafish	Arterial ECs (E11.5, 48 hpf)		44, 87
***Tal1/Scl* (*ms*)**	Scl+19	Downstream +19 kb	Tg mouse	EC^a^ (E11.5)	**MYB**: mo **ETS**: mo, em (FLI1/ELF1) **GATA**: mo, em (GATA2)	88
***Tal1/Scl* (*ms*)**	TAL1‐3.8	Upstream −4 kb	Tg mouse	Pan‐EC (E11.5)	**ETS**: mo, em (FLI1/ELF1), oe (FLI1/ELF1)	89
***Tie1* (*ms*)**	Tie promoter	Upstream/promoter −1 kb	Tg mouse	Pan‐EC (E8.5–E10.5) Organ specific (E15–E17) Large vessels (adult)	**ETS**: mo, mu, oe **AP1/2/4**: mo	90, 91, 92
***Tie2* (*ms*)**	Tie2 HHXK and Tie2 HHNS	Promoter/intronic +1 kb	Tg mouse	Pan‐EC (E9.5‐adult)	**FOXC/O**: mo, em, ch, oe/OE **ETS**: mo, em (ETS1), oe/OE (ETV2), mu	45, 93, 94, 95

*Note*: Enhancer location is given as approximate kb from TSS of assigned gene and species. Categories of experimental validation methods linking TFs to particular enhancers include mo (motif for TF identified within enhancer); ch (ChIP confirms TF binding); chs (ChIP‐seq confirms TF binding), em (electromobility shift assay confirms TF binding), mu/MU (mutation tested in vitro/in vivo confirms role of motif); KO/KD (knock‐out/knock‐down analysis confirms role of TF in enhancer regulation) and oe/OE (overexpression of TF in vitro/in vivo confirms role of TF in enhancer regulation).

^a^ Indicates multiple TSS, value given to first.

^b^ Indicates mutation did not include all available motifs for that TF.

This review will focus on data from mouse models of vasculogenesis and angiogenesis, which predominantly involves analysis of vascular development during embryonic and early postnatal growth. Vasculogenesis research focuses primarily on the E7.5–E9.5 embryo, whereas a variety of later embryonic timepoints have been used to study angiogenesis (with the E10–E12 hindbrain providing a useful angiogenic‐only vascular bed^7^, ^96^). Additionally, the post‐natal retina (from post‐natal days (P)4–7) has been a favored model for many angiogenic studies,^96^, ^97^ while angiogenesis can also be studied in the adult after insertion of Matrigel plug or tumor cells. These models are often used in combination with different Cre‐driver transgenes to delete floxed genes‐of‐interest, permitting endothelial specific (e.g., Tie2‐Cre), induced (e.g., RosaCreER) or induced endothelial specific (e.g., CDH5‐CreERT2 and PDGFB‐iCreERT2) patterns of gene deletion (reviewed by 98), although constitutive gene deletion has also been a useful approach for those genes largely specific to ECs. Because the efficacy of Cre and CreERT2 transgenes can vary considerably (both between different types of drivers, and between different versions of the same Cre transgene^98^), and tamoxifen administration alongside Cre can itself result in endothelial phenotypes,^99^ analysis of results should always consider the Cre driver utilized, the evidence of gene knockdown provided and the quality of controls. In vitro models can also provide powerful information (e.g., analysis of embryonic stem cell differentiation to study vasculogenesis^100^; angiogenesis assays using cultured ECs to investigate sprouting from EC spheroids^101^) and are included alongside animal studies where relevant.

## 2 ETS

ETS proteins comprise a large family of TFs characterized by a common DNA‐binding domain (the ETS domain) which mediates binding to a core GGA^A^/_T_ motif (reviewed by^102^ and summarised in Table 1). While ETS factors are not specific to ECs and contribute to the differentiation of other cell types (e.g., PU.1 in hematopoiesis,^103^ ELK1 in neurogenesis,^104^ see Table 2), it is increasingly appreciated that ETS factors play a key role in EC identity. In particular, ETS binding motifs are a defining and essential feature of all known endothelial‐expressed promoters and enhancers (e.g., 45, 48, 57, 58, 76 and Table 3). However, analysis of the direct role(s) of ETS TFs within the vasculature has been complicated by the sheer number of ETS family members expressed in ECs. Adult ECs express at least 13 different ETS factor genes (*Ets1*, *Ets2*, *Erg*, *Fli1*, *Etv3*, *Etv6/Tel*, *Elk1*, *Elk3/Net*, *Elk4/Sap1*, *Elf1*, *Elf2/Nerf*, *Elf4*, and *Gabpa*), although consistent high expression across adult aorta, lung and brain ECs is principally restricted to *Ets1*, *Ets2*, *Erg*, *Fli1*, *Elk3*, *Elf1*, *Elf2*, and *Gabpa*, the latter three of which are also highly expressed in many other cell types.^102^, ^105^, ^106^ Of these adult ETS, all but *Elk1* are also expressed in angioblasts in E8.0 embryos.^2^ Additionally, *Etv2 (Etsrp71)* is strongly expressed in hemato‐endothelial progenitors in the early mouse embryo,^107^ although little expression is reported after mid‐gestation. Similarly, *Fev*, *Etv4*, *Etv5*, and *Erf* are expressed just after *Etv2* in the same progenitor population,^2^, ^108^ although none are strongly expressed in adult ECs. Consequently, analysis of phenotypes after deletion of a single ETS factor must always consider the potential for functional redundancy and compensation. Nonetheless, gene deletional studies in both mouse and zebrafish have repeatedly found significant endothelial defects downstream of ETS factor deletion, strongly supporting a key role for ETS factors in the regulation of vasculogenesis and angiogenesis.^102^, ^109^ It is, however, unlikely that ETS factors regulate their gene targets alone: gene enhancers with entirely different patterns of EC expression (e.g., arterial‐specific, angiogenic‐specific, and vein‐specific) can all be robustly bound and activated by ETS factors (e.g., 32, 48, 49, 50, 51, 57, 69, 85; see also Table 3) indicating that ETS‐driven regulation of many aspects of endothelial behavior is likely to involve combinatorial interaction with other families of TFs to achieve the required spatio‐temporal specificity.

### 2.1 ETV2

Of all the ETS TFs investigated in endothelial knock‐out mouse models, only loss of ETV2 (ETSRP71/ER71) ablates vasculogenesis, resulting in a complete absence of both endothelial and blood cell lineages and subsequent lethality by E9.0.^61^, ^107^ Conversely, prolonged activation of *Etv2* leads to an overly dense capillary bed, hemorrhage, and an absence of hematopoietic cell differentiation.^110^ Predating these discoveries, knockdown of the zebrafish orthologue, *etsrp/etv2*, was also shown to result in cessation of embryonic circulation.^109^, ^111^

*Etv2* is expressed within a narrow time window during mouse development between E6.75 and E9.5 (Table 2), a transience that is crucial for endothelial maturation. It is first observed in a subset of cells in the posterolateral mesoderm at E6.75–7.0,^2^, ^108^, ^112^ with the strongest *Etv2* expression occurring in hemato‐endothelial progenitor populations rather than differentiated ECs by E8.5.^2^, ^19^, ^113^ While it is possible that the uniquely early expression pattern of *Etv2* contributes to the severe consequences of deletion, overexpression of *Fli1*, *Erg* and *Ets1* in ETV2‐null embryoid body culture systems could not fully rescue the formation of the endothelial lineage.^1^ Alternatively, the importance of ETV2 in the specification of ECs may, at least in part, be attributed to its crucial role in regulating the expression of *Flk1* (also known as *Kdr* and encoding the VEGFR2 receptor). In the mouse embryo, loss of ETV2 markedly reduces the number of VEGFR2+ cells,^114^ likely due to direct regulation of *Flk1* through ETV2 binding at *Flk1* enhancer elements^69^, ^115^, ^116^ (Table 3). While not all *Flk1* expression is lost, those VEGFR2+ cells that persist have a cardiac rather than EC lineage.^117^ This is supported by studies in embryonic stem cell cultures, which also found that cells differentiate into cardiac lineages in the absence of ETV2.^112^, ^114^, ^118^ ETV2 is therefore not essential for the expression of *Flk1* per se, but instead up‐regulates expression levels to create a subpopulation of high VEGFR2+ cells driven towards an endothelial fate.^1^ VEGFA signaling through VEGFR2 is a potent activator of MAPK/ERK signaling (reviewed in 119), which in turn phosphorylates ETS factors and increases their binding affinity (reviewed in 120, 121). Therefore, VEGFA‐VEGFR2 signaling enhances *Flk1* gene transcription through an ETV2‐dependent positive feedback loop.

In addition to *Flk1*, ETV2 is implicated in the direct activation of many other key endothelial lineage specifying genes, including *Cdh5*, *Tek*, *Tal1(SCL)*, *Notch4*, *Nfatc1*, and *Sox7*,^45^, ^114^, ^122^, ^123^ often but not always in combination with Forkhead TFs (see below and Table 3). Additionally, ETV2 can play a role in the activation of other endothelial ETS factors, including *Fli1*, *Ets1*, *Ets2*, *Erg*, and *Fev*.^1^, ^108^ It has been hypothesized that ETV2 may act as a pioneer factor, creating an endothelial lineage‐specific epigenetic landscape.^124^ Supporting this, ETV2 binding can directly result in demethylation of its binding sites, and this hypomethylation can be maintained in blood and vascular systems as an epigenetic memory.^1^, ^125^ Further, this role may be facilitated by complexing with TET1/TET2 enzymes and thereby directly promoting locus‐specific reversal of methylation marks.^126^ ChIP‐seq data show that binding sites at regulatory elements previously occupied by ETV2 become occupied by other ETS factors at later stages of development.^1^ Cultured amniotic cells can also transition into immature ECs through transient *Etv2* expression, while co‐expression with *Fli1* and *Erg1* is required for maturation.^118^ It is therefore clear that ETV2 plays a unique role in establishing EC fate during vasculogenesis, both specifying the endothelial epigenetic landscape and directly establishing the expression of lineage‐specifying genes that will maintain EC fate once ETV2 itself is gone.

### 2.2 FLI1

FLI1 is expressed in hemato‐endothelial progenitors soon after ETV2 and remains highly expressed in ECs throughout development (see Table 2). However, unlike *Etv2*, constitutive deletion of *Fli1* in mice does not affect vasculogenesis, although *Fli1* null mice die at E11 due to cerebral hemorrhage and loss of vessel integrity. Instead, FLI1 may play a role in the differentiation of angioblasts into a functional vascular network,^127^, ^128^ and has been implicated in the direct regulation of many genes involved in maintaining vascular homeostasis (including *Cdh5*, *Cd31*, *Col4a1*, *Mmp9*, *Pdgfb*, and *S1pr1*).^129^ However, endothelial‐specific deletion of *Fli1* mediated by Tie2‐Cre results in a milder phenotype and mice are born at expected mendelian ratios.^129^ While this could indicate an EC‐independent role for FLI1 in vascular assembly, it is also possible that critical EC functions of FLI1 occur before the onset of Tie2‐Cre activity. Further, the limited phenotype may be explained by functional redundancy between FLI1 and the homologous ETS factor ERG. ERG shares over 70% amino acid sequence similarity and a near‐identical ETS domain with FLI1,^130^ but is expressed slightly later during development.^2^, ^19^, ^131^ Of note, it has recently been shown that induced endothelial‐specific compound knockdown of *Fli1* and *Erg* together in adult mice results in rapid lethality alongside transcriptional silencing of core EC genes, strongly indicating both significant functional redundancy and a shared yet crucial role for FLI1/ERG in EC identity.^132^

### 2.3 ERG

ERG is the most highly expressed ETS factor in the mature mouse vasculature, with robust EC expression beginning at E7.75–8.0 and maintained throughout the lifespan across all EC subtypes^2^, ^133^, ^134^, ^135^ (Table 2). Although highly expressed in ECs, ERG is not endothelial‐specific, with expression also observed in some blood cell lineages, in osteoblasts and in chondrocytes (reviewed by 136). Endothelial‐specific deletion of *Erg* leads to growth defects, cardiovascular abnormalities, hemorrhage, and embryonic lethality at E10–E12.5, indicating a crucial and independent role for ERG in vascular development.^137^, ^138^ Analysis of ERG binding patterns in cultured ECs suggests that it promotes endothelial homeostasis via directly binding a majority of active endothelial enhancers and super‐enhancers.^33^ Such genome‐wide enhancer occupancy underscores the critical role ERG plays in the regulation of endothelial function, although unlike ETV2, ERG enhancer occupancy requires a pre‐specified chromatin landscape.^33^

ERG is also known to directly regulate many pro‐angiogenic genes, including those involved in EC migration, apoptosis, and vascular stability (reviewed in 136). VEGF‐VEGFR2 signaling during angiogenesis can induce phosphorylation and activation of ERG via the MAPK/ERK pathway, resulting in increased ERG binding and co‐factor recruitment at regulatory elements driving angiogenic gene expression (e.g., *Dll4* and *Hlx* ^49^), although this VEGF‐enriched ERG binding is non‐specific and is equally found at arterial and venous‐specific enhancers.^57^ Additionally, siRNA‐mediated knockdown of ERG reduces vascularization of Matrigel plugs, a phenotype linked to reduced *Hdac6* expression.^139^, ^140^ ERG‐deficient ECs also have reduced WNT signaling, with ERG influencing angiogenesis by promoting β‐catenin stability via the Wnt receptor FZD4 and CDH5 (VE‐Cadherin).^137^

### 2.4 ETS1/2

*Ets1*, the founding member of the ETS family, is first seen in hemato‐endothelial progenitors from E7.0 to E7.25 and is strongly expressed in mature endothelium as well as in other tissues^2^, ^141^, ^142^ (Table 2). Like several other non‐EC ETS factors, ETS1 exhibits autoinhibition (meaning the full‐length protein has less affinity for DNA than the binding domain alone), which is reinforced by phosphorylation and counteracted by protein partnerships.^130^ ETS1 is expressed in ECs at the sites of both developmental and tumor angiogenesis,^143^, ^144^, ^145^ and is up‐regulated by both pro‐angiogenic VEGF‐VEGFR2 and hypoxia signaling pathways.^146^ Although constitutive deletion of *Ets1* results in few vascular defects, ETS1 shows significant functional redundancy with its close homolog ETS2, with which it shares a PNT domain targeted by Raf/Mek/Erk‐mediated phosphorylation.^130^ While single knockout of *Ets2* also does not result in significant vascular phenotypes, compound *Ets1; Ets2* knockout mice are embryonic lethal by E12 with dilated vessels, failed blood vessel branching, edema, and hemorrhage, suggesting a key but redundant role for ETS1/2 in angiogenesis.^113^ Dominant negative ETS1 also inhibits retinal angiogenesis during proliferative retinopathy,^145^ while antisense oligonucleotides directed against ETS1 inhibit EC migration and VEGF‐induced EC proliferation.^144^ The many characterized EC direct target genes include *Flt1*, *Tie2 (Tek)*, *Angpt2*, *Nrp1*, *Vwf*, *Cd31*, *Cdh5*, and crucially *Flk1*.^90^, ^142^, ^147^, ^148^, ^149^, ^150^ ETS1 binding is enriched at gene promoters, and strongly correlates with transcription^23^: in cultured ECs, VEGFA stimulation results in increased ETS1 occupation at both P300‐bound enhancers and promoters of activated angiogenic genes.^23^, ^151^ High expression of ETS1 in EC lines can also drive the switch from a quiescent to angiogenic state, which is attributed to increased expression of matrix metalloproteinases.^113^, ^152^

### 2.5 ELK3

Among the ETS factors expressed strongly and early in ECs, the function of ELK3 (NET) is probably the least understood. ELK3 is part of the ternary complex factor (TCF) subfamily of ETS alongside ELK1 and ELK4 (SAP1), all of which contain an additional B‐box domain to mediate interaction with serum response factor (SRF). *Elk3* expression is first seen in hemato‐endothelial progenitors from E7.0 (concurrent with *Fli1* but after *Etv2)* and persists in ECs throughout development and in the adult^2^, ^153^, ^154^ (Table 2). In the absence of MAPK activation (particularly isoforms ERK2, JNK, or p38), ELK3 strongly inhibits transcription,^130^ and the switch from inhibitor to activator has been implicated in the role of ELK3 in angiogenesis.^155^ However, while tumor cells with reduced ELK3 form fewer vessels, and *Elk3* down‐regulation inhibits VEGFA expression^155^ and modulates HIF1 stability,^156^ constitutive *Elk3* deletion results in viable mice with only mild vascular defects.^157^ Compound deletion of orthologues *Elk1* and *Elk4* also fail to show angiogenic defects, leading to the conclusion that the crucial role of SRF in angiogenesis occurs via alternative MRTF co‐factors.^158^, ^159^, ^160^ While no true *Elk1;Elk3;Elk4* triple knockout mice have been studied, neither *Elk1* or *Elk4* are strongly expressed during early endothelial development, and consequently the precise role of ELK3, and of the TCF ETS subfamily, in vasculogenesis and angiogenesis is still not fully defined.

## 3 FORKHEAD

Forkhead (FOX) TFs are characterized by a winged‐helix Forkhead box DNA binding domain which binds a core ^C^/_T_AAA^C^/_T_A motif (Table 1). The 44 different FOX proteins found in mice and humans can be divided into 22 subclasses (denoted as FOXA through to FOXS) according to sequence similarities,^161^ with members of the FOXC and FOXO subfamilies the principal FOX factors implicated in the regulation of vasculogenesis and angiogenesis.

### 3.1 FOXC1/2

Both *Foxc1 (Mf1)* and *Foxc2 (Mfh1)* are expressed in ECs from early in embryonic development, detected by scRNA‐seq from around E8.0 and by in situ hybridization from E9.5^2^, ^162^ (Table 2). Compound *Foxc1;Foxc2* deletion results in a failure of blood vessel development and lethality by E9.5. Although ECs are specified and an initial vascular plexus forms in these mutant mice, the plexus does not remodel into a functional vascular network,^162^, ^163^ indicating important functions for FOXC factors in endothelial differentiation and angiogenesis. In particular, FOXC factors have been implicated in angiogenesis via direct induction of *Itgb3*‐mediated EC adhesion and migration,^164^ and of *Dll4*‐mediated Notch signaling downstream of VEGF.^165^, ^166^ Analysis of endothelial enhancers also identified a shared role for FOXC and ETS factors in endothelial specification. FOXC1/2 and the ETS factor ETV2 combinatorially bind compound FOX:ETS motifs within gene enhancers^167^ and promoters to synergistically activate the transcription of crucial endothelial lineage‐identity genes, including those for *Flk1*, *Flt4*, *Tal1*, *Cdh5*, *Tie2*, *Notch4*, and *Pdgfrβ* ^45^ (see Table 3). Further, FOXC factors are also important regulators of endothelial patterning in the maturing vasculature. Embryos in which only a single FOXC allele remains show severe arterio‐venous malformations and lack expression of arterial/angiogenic‐associated genes including *Notch1*, *Dll4*, and *Jag1*,^163^ while lymphatic‐specific compound deletion of *Foxc1*;*Foxc2* results in increased lymphatic EC proliferation and abnormal lymphatic vessel morphogenesis.^168^

### 3.2 FOXO1/3/4

Mammalian *Foxo1 (Fkhr)*, *Foxo3 (Fkhrl1)*, and *Foxo4 (Afx)* encode the evolutionarily conserved FOXO subfamily, which act as key nuclear effectors of the PI3K/AKT pathway. In the absence of active PI3K/AKT signaling, FOXOs localize to the nucleus, while PI3K activation results in FOXO phosphorylation and subsequent nuclear exclusion and proteasomal degradation.^169^, ^170^, ^171^ FOXO1, the most robustly expressed *Foxo* gene in ECs (Table 2), is expressed from E7.75 and plays a crucial role in vascular development in both the embryo and the adult: constitutive and EC‐specific deletion of *Foxo1* results in a failure of angiogenesis‐dependant vascular remodeling and lethality by E10.5.^172^, ^173^, ^174^ It is also able to bind the compound FOX:ETS motifs found in many early EC enhancers.^45^ *Foxo3a* null and *Foxo4* null mice do not die during embryogenesis; however, post‐natal angiogenic capacity is increased in *Foxo3a* knockout mice.^175^ FOXO1 is also required to direct angiogenesis in the post‐natal retina, with deletion resulting in uncoordinated vascular growth, increased endothelial number, density, and vessel diameter.^174^ Mechanistically, FOXO factors function by coupling changes in metabolism with changes in gene transcription and cell activity. In ECs, the transcriptional targets of FOXOs include antioxidants, cell cycle inhibitors, and metabolic regulators, and they act as potent negative regulators of MYC activity.^174^ FOXO activity itself is also regulated by energy‐sensing post‐transcriptional modifiers.^176^ Therefore, in ECs, FOXO1 activity reduces metabolic activity, reduces MYC‐induced glycolysis and mitochondrial respiration, thus mediating angiogenesis in response to changes in metabolism.^174^

### 3.3 Other FOX factors

*Foxm1* is ubiquitously expressed in the early embryo but may play a role in vascular growth. Although EC‐specific deletion of *Foxm1* results in no overt phenotype, pulmonary vascular injury in mice lacking or overexpressing *Foxm1* revealed a role for EC FOXM1 in the restoration of endothelial barrier function.^177^, ^178^ Additionally, both *Foxp1* and *Foxp4* are expressed during early endothelial development,^2^ with *Foxp1* most highly expressed in ECs in culture, and up‐regulated in ECs during injury‐induced neovascular growth. Knockdown of *Foxp1* in ECs in culture inhibits EC proliferation, migration, and tube formation,^179^ while constitutive *Foxp1* knockout mice die at E14.5 with complex cardiovascular defects including vascular hemorrhage.^180^ However, the vessel defects are thought to be secondary to severe cardiac and valve defects and were not seen after EC‐specific *Foxp1* deletion.^181^

## 4 GATA

The GATA family consists of six TFs characterized by a highly conserved zinc finger domain binding a core GATA motif (reviewed by 182; Table 1). GATA1, GATA2, and GATA3 have well‐established roles in the specification of hematopoietic cells from the hemogenic endothelium (a specialized subset of ECs that give rise to the hematopoietic stem and progenitor cells, reviewed by 183), but are also implicated in EC specification and development.

### 4.1 GATA2

*Gata2* is expressed in hemato‐endothelial progenitors from E7.0 (Table 2) and has been implicated in the regulation of EC lineage specification via interaction with ETV2.^184^ Motif analysis of endothelial enhancers also supports a role for GATA2 in the expression of angiogenic genes, with all characterized *Flk1* EC enhancers containing essential GATA‐binding motifs^69^, ^185^ (Table 3). GATA2 binding at the *Flk1* promoter also increases expression in response to changes in matrix stiffness,^186^ while expression of *Emcn* (Endomucin), a type I integral membrane glycoprotein important in EC signaling and angiogenesis, requires GATA2‐mediated chromatin remodeling.^187^ Furthermore, GATA2 binds a unique set of gene loci in ECs compared to other cells lines,^28^ dynamic GATA2 binding has been observed at key EC enhancers in response to VEGFA stimulation,^31^ and siRNA‐mediated GATA2 knockdown in the post‐natal retina inhibits angiogenic sprouting.^186^ However, constitutive deletion of *Gata2* had no clear effect on early vascular formation before embryonic lethality at E10.5 due to hematopoietic defects,^188^ although it is possible that the deletion strategy used to generate these mice (which removes only the C‐terminal zinc finger of GATA2) did not ablate all GATA2 DNA binding.^189^ Alternatively, the early hematopoietic lethality may predate overt EC phenotypes. Supporting this, both constitutive deletion of the GATA2+9.5 enhancer (strongly active in developing embryonic ECs)^72^ and endothelial‐specific deletions of *Gata2* result in lethality at E13–16.5 with vascular abnormalities including hemorrhage, edema, and anemia. However, important roles for GATA2 in lymphangiogenesis underpins many of these defects.^85^, ^190^

GATA3 and GATA6 are also expressed in ECs in culture and have been implicated in the regulation of *Tie2*.^191^ Although it is possible that endothelial GATA2 may be functionally redundant to some degree with these other GATA factors, there is little evidence that GATA3 or GATA6 are robustly expressed in the developing endothelium (Table 2). Consequently, the precise roles of GATA factors in vasculogenesis and angiogenesis remain unclear.

### 4.2 GATA/TAL1

Combinatorial binding of GATA alongside the TAL1/SCL TF at a composite GATA‐E‐box binding motif plays a key role in hematopoietic development.^192^, ^193^ In particular, GATA/TAL1 binding motifs are essential for the maintenance of hematopoietic stem cells and for the terminal differentiation of select blood cell lineages.^192^, ^193^, ^194^ GATA/TAL1 binding may also play a role in maintaining endothelial identity in the early embryo: constitutive *Tal1* knockout embryos display irregular yolk sac vasculature,^195^, ^196^ while EC‐specific deletion of *Tal1* results in edema, hemorrhage, and defective vascular remodeling in the yolk sac.^197^ These defects have been attributed to dual roles for TAL1, in both activating hemogenic endothelium and repressing ectopic cardiomyogenesis in the yolk sac endothelium and endocardium.^198^, ^199^ ChIP‐seq experiments that assess chromatin states of enhancer elements suggest that TAL1 exploits a pre‐established epigenetic landscape, likely generated by ETV2, to bind and repress enhancers of the cardiac lineage, while binding together with GATA to activate endothelial/hematopoietic lineage specification.^200^

## 5 SMAD

SMAD proteins are the transcriptional effectors of the TGFβ superfamily and can be subdivided into receptor‐regulated SMADs (R‐SMADs, consisting of SMAD1, 2, 3, 5, and 8), common SMAD (SMAD4), and inhibitory SMADs (I‐SMADs, SMAD6 and 7).^201^, ^202^ R‐SMADs act as the primary signal transducers, forming complexes with SMAD4 after phosphorylation and subsequently translocating to the nucleus to directly regulate gene transcription.^202^ R‐SMADs can be further subdivided into those primarily downstream of canonical BMP signaling (SMAD1/5/8) and those primarily downstream of canonical TGFβ signaling (SMAD2–3). Both BMP and TGFβ signaling pathways play complex and sometimes contradictory roles in angiogenesis. TGFβ signaling via ALK1 and endoglin can stimulate EC activation, proliferation, and migration, whereas TGFβ signaling via ALK5 can inhibit proliferation and promote vascular maturation (reviewed by 203). Similarly, BMP signaling is implicated in both the promotion of angiogenesis and the maintenance of endothelial homeostasis via interactions of different ligands, receptors, and co‐factors (reviewed by 202). Most SMADs are expressed early during EC development but also widely in non‐ECs (Table 2). While endothelial‐specific deletion of *Smad2/3* did not affect vasculogenesis and early angiogenesis (although defects in vascular maturation and mural cell assembly resulted in hemorrhage and lethality by E12.5), endothelial‐specific deletion of either *Smad4* or *Smad1/5* led to defective angiogenic sprouting and embryonic lethality by E10.5.^204^, ^205^ Analysis of SMAD1/5 binding in ECs identified numerous direct targets, including *Id1*, Notch pathway genes *Hey1*, *Hey2*, *Hes1*, and *Jag1*, and venous identity genes *Ephb4* and *Coup‐TFII (Nr2f2)* ^20^, ^58^, ^204^ (Table 3). These targets have also been verified in knockout models, with endothelial‐specific deletion of *Smad4* resulting in embryos with reduced *Ephb4* and *Coup‐TFII* expression and defective venous differentiation, while endothelial‐specific deletion of *Smad1/5* resulted in embryos with impaired DLL4‐Notch signaling. The inhibitory SMAD6 may also play a role in this process downstream of Notch and upstream of target gene activation.^206^ Additionally, analysis of retinal angiogenesis after postnatal deletion of *Smad4* found increased EC proliferation and distorted arteriovenous gene expression, implicating SMADs in the pathogenic formation of arteriovenous malformations downstream of BMP9/10‐ALK1 and upstream of angiopoietin‐Tek signaling.^207^, ^208^, ^209^

## 6 HYPOXIA‐INDUCIBLE FACTOR

Hypoxia‐inducible factor (HIF) TFs are master regulators of oxygen homeostasis^210^ and therefore have significant direct and indirect influences on vascular growth. HIF1α and HIF2α (EPAS) subunits are stabilized in low oxygen conditions, permitting them to translocate to the nucleus and bind DNA at consensus ^A^/_G_CGTG motifs (Table 1) as a heterodimer with HIF1β (ARNT), inducing gene programs that respond to the hypoxic environment (reviewed by 211). This adaptive response includes the activation of physiological and pathological angiogenesis through both cell autonomous and non‐autonomous mechanisms.^212^, ^213^ While *Hif1a* is ubiquitously expressed, *Hif1b* is predominantly EC‐specific from E8.5 (see Table 2) where it is up‐regulated by hypoxia^214^, ^215^ (Table 2). Fewer than 20% of HIF target genes are regulated by both isoforms in ECs indicating predominantly non‐overlapping functions, with HIF2α inducing a larger and more diverse transcriptional response.^216^ Overexpression of HIF isoforms in cultured ECs increases expression of a range of pro‐angiogenic genes, including *Vegfa*, *Angpt2*, and *Pdgfb* (by HIF1α)^217^, ^218^, ^219^ and *Flt1* (by HIF2α).^220^ However, studies into the role of HIFs in ECs during developmental angiogenesis in mice have produced conflicting results. EC‐specific expression (driven by a *Flk1* enhancer/promoter) of a dominant negative HIF mutant (which inhibits transcriptional responses by both HIF1α and EPAS1/HIF2α) results in embryonic lethality by E11.5 alongside severe cardiovascular defects and loss of vascular sprout formation attributed to reduced *Tie2 (Tek)* expression.^221^ Embryonic lethality and severe vascular defects (with reduced *Tie2*) were also seen after constitutive deletion of *Hif2a*.^222^ However, *Cdh5‐Cre*‐driven EC‐specific deletion of *Hif2a* alone or in combination with *Hif1a* resulted in no major developmental vascular abnormalities,^223^, ^224^ although increased vascular permeability and pulmonary hypertension were seen in the adult.^224^ This discrepancy in phenotype may be due to an early role for HIFs before robust *Cdh5‐Cre* activity or incomplete Cre‐mediated deletion (in part because the Cdh5‐Cre driver used here is known to be only weakly and sporadically expressed before E14.5,^98^, ^225^ whereas the Flk1 driver of dominant negative HIF is active more robustly and much earlier), or may instead indicate a key function for HIF proteins in the activation of *Vegfa* in non‐EC lineages.^226^, ^227^ Alternatively, off‐target effects of the dominant negative form of HIF may have contributed to the severity of phenotype seen in the dominant negative mutant mouse.

## 7 RBPJ

The RBPJ (CSL) TF acts as the nuclear effector of the Notch signaling pathway, forming a complex with Notch intracellular domain (NICD) and the MAML1 co‐activator to promote transcription via direct binding to Notch target genes at a consensus TGGGAA motif (reviewed by 228 and Table 1). Analysis of many deletional models strongly indicates that Notch signaling via RBPJ plays an essential role in vasculogenesis, angiogenesis, and arteriovenous differentiation.^8^, ^9^, ^229^, ^230^ Loss of multiple different Notch pathway components results in early lethality in mice associated with severe vascular defects including aberrant arteriovenous specification (e.g., compound *Notch1/Notch*4 deletion,^231^ deletion of ligand *Dll4* ^232^, ^233^), while ablation of Notch signaling in the post‐natal retina results in significant defects in sprouting angiogenesis (reviewed by 8, 10). Loss of RBPJ largely recapitulates defects downstream of Notch receptor/ligand deletion,^9^, ^232^, ^234^ and the Notch signaling components *Hey1* and *Dll4* are both direct targets of RBPJ via arterial/angiogenic‐specific enhancers.^50^, ^75^ Further, multiple different signaling pathways can converge upon, and influence, RBPJ binding. For example, Ang1/Tie2 signaling induces *Dll4* via AKT‐mediated activation of β‐catenin, which complexes with RBPJ to bind and activate an intronic enhancer,^52^, ^235^ whereas KLF4 can inhibit *Dll4* expression during angiogenesis by interfering with RBPJ binding to the same element^53^ (Table 3). In the absence of NICD, RBPJ can also act as a transcriptional repressor, and may repress EC gene expression in some circumstances. For example, RBPJ binding to an *Flk1* arterial enhancer represses its activity in veins,^69^ while RBPJ binding to a *Vegfa* promoter negatively regulates its expression.^9^ However, although it was long assumed that Notch‐RBPJ influenced vascular patterning by directly regulating key arteriovenous genes, postnatal deletion of RBPJ does not ablate arterial gene expression,^234^, ^236^ and ECs lacking MYC (a key driver of metabolism and proliferation) require neither RBPJ nor Notch for correct arteriovenous gene expression.^237^ Consequently, it is now thought that Notch‐RBPJ regulates arteriovenous specification by reducing metabolism and cell cycle rather than via direct activation or repression of arteriovenous identity genes.^237^ Given that the signaling processes involved in angiogenic sprouting are often coupled to arterial formation,^9^ it is still unclear to what degree RBPJ directly versus indirectly regulates gene expression in arterial and angiogenic ECs.

## 8 HEY FACTORS

The Hey family of transcriptional repressors are basic helix‐loop‐helix proteins that act downstream of Notch signaling and are directly activated by RBPJ^75^, ^238^ (Table 3). *Hey1* and *Hey2* are expressed in ECs from early during mammalian development (see Table 1) and directly bind DNA at E‐box motifs.^238^ While the orthologue of *Hey2* in zebrafish (*gridlock*) is essential for arterial morphogenesis,^239^ deletion of either *Hey1* or *Hey2* separately does not result in gross embryonic vascular phenotypes, although *Hey2* null mice die soon after birth and *Hey1* null mice show anomalies of the thoracic great vessels.^240^, ^241^ However, compound deletion of both *Hey1* and *Hey2* results in embryonic lethality and significant endothelial defects including in arteriovenous differentiation.^242^, ^243^ Loss of HEY1/HEY2 resulted in both reduced *Ephb2* and *Jag1* expression and increased *Robo4* and *Flk1* expression.^242^, ^243^, ^244^ However, there are currently few well‐characterized direct endothelial targets of HEY1/2 described in the literature, and it is unclear to what extent the phenotype in the compound *Hey1/2* null mice can be attributed to vascular versus cardiac defects.^242^, ^243^, ^244^

## 9 HLX

HLX1 is a homeobox TF with a currently undefined DNA binding motif expressed in hemato‐endothelial progenitors from E7.75 (Table 2). HLX1 was first implicated as a key regulator of sprouting angiogenesis in zebrafish (as the orthologue *hlx*), where it maintains endothelial stalk‐cell fate in a cell‐autonomous manner.^245^ In mammalian EC culture, HLX1 regulates expression of cell guidance molecules including *Unc5b*, *Plxna1*, and *Sema3g* downstream of VEGF‐VEGFR2 signaling,^246^ and is a direct transcriptional target of both ERG and MEF2 TFs downstream of VEGFA‐VEGFR2 signaling.^49^, ^51^ However, although both overexpression and knockdown of *Hlx1* in cultured ECs disrupt sprouting angiogenesis,^246^, ^247^ constitutive *Hlx1* deletion in mice causes only a mild vascular remodeling phenotype.^247^ Consequently, the precise role and direct transcriptional targets of HLX1 in angiogenesis are yet to be clearly elucidated.

## 10 MEF2

The MEF2 family of TFs is characterized by their highly conserved MADS‐box and MEF2 domains which mediate dimerization, co‐factor interactions, and DNA binding to a consensus ^C^/_T_TA ^A^/_T_^A^/_T_^A^/_T_^A^/_T_ TA^A^/_G_ motif.^248^ They are found in multiple cell lineages and play key roles in a diverse range of developmental processes.^248^ While constitutive *Mef2c* deletion results in both cardiac and vascular defects,^249^, ^250^ endothelial‐specific *Mef2c* deletion has no effect on embryonic vascular development regardless of Cre driver,^251^, ^252^ although subtle defects are detected in pathological angiogenesis.^251^ However, *Mef2a*, *Mef2c*, and *Mef2d* are all expressed in the early endothelium^2^, ^51^ (Table 3), and studies from other cell types show strong functional redundancy between the three proteins.^253^

Analysis of direct MEF2 targets in ECs alongside combinatorial gene deletion has strongly implicated MEF2 factors in angiogenesis downstream of VEGFA‐VEGFR2 signaling, and in endothelial homeostasis downstream of blood flow.^51^, ^254^ Supporting this, induced EC‐specific deletion of *Mef2a* alongside *Mef2c* reduces sprouting angiogenesis and *Dll4* expression in the post‐natal retina, while MEF2 proteins directly bind and activate many angiogenic‐specific genes including *Dll4* and *Hlx1*.^51^ Further, compound endothelial‐specific deletion of *Mef2a*, *Mef2c*, and *Mef2d* in adult mice results in systemic inflammation, hemorrhage, and rapid lethality,^254^ closely phenocopying that of *Klf2/4* deletion and supporting a key role for MEF2 factors directly upstream of *Klf2/4* transcription in response to fluid shear stress.^254^, ^255^, ^256^ MEF2 factors also directly regulate the expression of *Mmp10* to regulate vascular integrity.^257^ It is, however, unclear how the widely expressed MEF2 factors are able to regulate such disparate vascular behavior. Although MEF2 factor activity can itself be regulated by both ERK5 and by complexing with class IIa HDACs, it is likely that additional transcriptional cofactors are required to enable MEF2 factors to achieve their diverse gene expression patterns in the endothelium.

## 11 AP1

AP1 proteins are made up of a ubiquitously expressed family of transcription complexes most commonly defined as a collection of dimers from the Jun and Fos families, although it can also be considered to include members of the ATF and MAF subfamilies.^258^ AP1 TFs bind a consensus TGA^C^/_G_TCA motif, although the ATF and MAF subfamilies have slightly differing preferences (see Table 1).

### 11.1 JUNB

Induced EC‐specific deletion of *Junb* in the retina leads to reduced angiogenic vascular growth and diminished expression of neurovascular guidance genes including *Sema3a*, likely via direct binding at AP1 motifs within enhancer elements.^259^ Although often considered ubiquitous, *Junb* expression is enriched in hemato‐endothelial progenitors and angioblasts in the E8.5 embryo.^2^ Further, JUNB is spatially restricted at the angiogenic front by a combination of VEGFA‐VEGFR2 and S1P‐S1PR signaling: induction of *Junb* by VEGFA‐VEGFR2 is countered by the circulating vascular maturation factor sphingosine 1‐phosphate (S1P), which restricts *Junb* expression in the perfused vasculature via S1PR‐dependent VE‐cadherin assembly.^259^ The related factor JUN/c‐JUN may also play a role in angiogenesis in some contexts and is implicated in the regulation of expansion of the vasa vasorum downstream of extracellular ATP signaling.^260^

### 11.2 MAFB

MAFB, a member of the large MAF TF subfamily, has been implicated in the regulation of both lymphangiogenesis and sprouting angiogenesis.^261^, ^262^ Analysis of the actively translated transcriptome at different stages of postnatal retinal angiogenesis combined with promoter analysis identified MAFB as a key regulator of postnatal angiogenesis.^261^ *Mafb* is enriched at the angiogenic front in postnatal retinas, while induced EC‐specific deletion results in defective angiogenic expansion. MAFB expression up‐regulates *Git1* and down‐regulates *Arhgdib* expression, two Rho GTPase modulators that activate and inhibit Rac1/Cdc42 signaling, respectively, leading to cytoskeletal changes and EC migration during sprouting angiogenesis.^261^ However, constitutive deletion of *Mafb* in mice results in no vascular‐related lethality and embryonic angiogenesis is unaffected,^263^ suggesting organotypic specificity of its angiogenic role or potential redundancy. In lymphatic ECs, *Mafb* expression is strongly up‐regulated by VEGFC, while *Mafb* overexpression results in increased levels of many lymphatic EC genes including *Prox1*.^264^ Additionally, although viable both constitutive and lymphatic EC‐specific *Mafb* deletion results in impaired lymphatic patterning, further supporting a role for MAFB in lymphatic ECs.^264^, ^265^

### 11.3 Small MAFs

Analysis of the epigenetic and transcriptional changes in cultured ECs following VEGFA stimulation identified small MAF proteins MAFF, MAFG, and MAFK as key regulators of the angiogenic transcriptional response alongside ETS1, ERG, MEF2C, and FOXO1.^31^ This is supported by in vitro sprouting angiogenesis assays, which indicate partially redundant but collectively critical functions for small MAFs in EC migration, proliferation, and tube formation.^31^ However, these observations have yet to be validated in animal models.

## 12 TEAD

The TEAD/TEF TF family consists of four highly homologous proteins (TEAD1–4), all containing the conserved TEA DNA‐binding domain recognizing a consensus GGAATG motif^266^ (Table 1). TEAD proteins are the mediators of YAP/TAZ‐dependent gene regulation downstream of the highly conserved Hippo signaling pathway. In their active state, YAP and TAZ translocate to the nucleus and form YAP/TAZ‐TEAD complexes, which are associated with the expression of genes controlling cell proliferation, migration, and apoptosis.^267^ YAP/TAZ activity is limited through phosphorylation by Hippo pathway components, leading to cytoplasmic retention and destabilization.^268^

EC‐specific deletion of *Yap/Taz* in mice leads to embryonic lethality associated with severe vascular defects throughout the embryo and yolk sac.^269^, ^270^ Similarly, induced EC deletion of *Yap/Taz* in the postnatal retina results in reduced vessel growth, blunted‐ended tip cells with fewer filipodia and defective lumen formation.^269^ Lowered levels of tip cell‐associated ANGPT2 and ESM1 are observed, the number of ERG‐positive ECs is reduced, and fewer actively proliferating ECs are detected.^269^ Conversely, overexpression of a stabilized TAZ protein results in a dense and hyperplastic vascular network with increased EC proliferation.^271^ Notably, induced EC‐specific compound deletion of *Tead1*, *Tead2*, and *Tead4* together results in a similar phenotype to that seen after *Yap/Taz* deletion, validating TEADs as crucial transcriptional effectors of endothelial YAP/TAZ signaling.^271^ Potential mechanisms for YAP/TAZ/TEAD involvement in angiogenesis include activation of actin cytoskeleton remodeling downstream of VEGF‐VEGFR2,^269^, ^270^ modulation of MYC signaling,^269^ activation of the small GTPase CDC42,^272^ and fuelling nutrient‐dependent mTORC1 signaling via transcriptional activation of cell‐surface transporters.^271^

## 13 SOXF

The SOXF proteins, comprising SOX7, SOX17, and SOX18, are the only members of the SOX TF family strongly expressed in the developing endothelium, and recognize a consensus ^A^/_T_CAA^A^/_T_ DNA motif.^273^ *Sox7* and *Sox17* are expressed concurrently with *Etv2* in angioblasts from the onset of vasculogenesis at E7.5, with *Sox18* also expressed in endothelial progenitors from E7.75.^2^, ^123^, ^274^, ^275^, ^276^ While SOX17 has been specifically implicated in arterial differentiation^277^ and SOX18 plays a key role in the initiation of lymphangiogenesis,^278^ SOXF factors also play crucial, although redundant, roles in vasculogenesis and angiogenesis. Although *Sox7* deficiency does not impact the emergence of ECs, vasculogenic defects to dorsal aorta formation are visible by E8.5, impaired angiogenesis is seen from E9.5, and both constitutive and EC‐specific deletion of *Sox7* results in significant growth retardation, severe vascular defects, and lethality by E10.5–E11.5.^273^, ^274^ EC‐specific deletion of *Sox17* results in a similar loss of angiogenic sprouting in some mouse backgrounds,^279^ as does compound heterozygous deletion of *Sox7* alongside *Sox17*.^273^ Complicating analysis, genetic levels of compensation between SOXF factors can vary depending on mouse background.^280^ This is evident in analysis of the role of SOXF factors in postnatal retinal angiogenesis, with the severity of single or compound deletion of *Sox7*, *Sox17*, and *Sox18* varying between mouse models. Notably, however, the resultant phenotypes are similar, resulting in ablation of tip and stalk cell identity, reduced vascular outgrowth and branching, and compromised tube formation and perfusion.^273^, ^281^ Overexpression of *Sox17* also promotes tumor angiogenesis and vascular abnormalities, while *Sox17* deletion in ECs reduces tumor angiogenesis and normalized tumor vessels, inhibiting tumor growth.^282^

The known direct SOXF target genes align with the range of vascular functions associated with these TFs. SOX17 directly binds arterial enhancers regulating *Dll4*, *Notch1*, and *Ece1*, while SOX18 directly targets a *Prox1* enhancer/promoter.^50^, ^56^, ^277^, ^278^, ^283^ SOX7 and/or SOX17 can also directly up‐regulate angiogenic *Flk1*,^69^, ^273^ *Lef1*, and *β‐catenin* ^284^ expression, while enforced expression of *Sox17* in EC‐like cells increases expression of *Col18a1* and *Cd31* as well as *Flk1*.^285^ However, given the wide and overlapping expression patterns of each SOXF factor both within and beyond the endothelium, it is apparent that additional co‐factors must be involved in the regulation of genes downstream of SOXF, while expression of the *SoxF* genes is themselves likely to be controlled by multiple upstream inputs.

## 14 FUTURE DIRECTIONS

Our understanding of the array of TFs involved in vasculogenesis and angiogenesis has greatly increased in the last ten years, as has our ability to link these factors both to specific aspects of EC biology and to their direct target genes. Progress in these areas will continue as new technologies increasingly provide information of gene expression patterns, enhancer marks, and protein binding patterns at a single‐cell resolution, and as computational pathways are developed to process and analyze such complex information simply and efficiently. Alongside this progress sit innovations in our ability to experimentally examine the role of novel EC transcriptional regulators, including the increasing ease of genetic manipulation, the generation of more diverse and specific methods to alter gene expression selectively in certain ECs, and higher‐throughput methods to validate and interrogate enhancer elements directly in animal models. However, while such approaches will improve our knowledge of the roles of known vascular TFs and identify new factors, a better understanding of gene regulation within the vasculature also requires a greater appreciation of the manner in which the limited cohort of TFs work together to achieve different outputs. As is made clear in this review, ECs contain no single lineage‐defining TF. Instead, the vast majority of endothelial transcriptional regulators are both expressed outside of ECs and involved in activating genes with more than one type of endothelial expression pattern and/or in response to more than one stimulus. Consequently, while improved genetic information and animal models will provide a more complete picture of the TF repertoire coordinating vasculogenesis and angiogenesis, this must be coupled with a greater understanding of the different combinatorial, synergistic, and antagonistic ways in which these factors work together to enable this limited number of proteins to achieve such complex and responsive patterns of gene expression.

## AUTHOR CONTRIBUTIONS

**Sophie Payne:** Writing – original draft (equal). **Alice Neal:** Writing – original draft (equal). **Sarah De Val:** Writing – review and editing (lead).
